# Rabbit targeted genomic sequences after heterologous hybridization using human exome

**DOI:** 10.1186/s13104-022-06162-5

**Published:** 2022-08-19

**Authors:** Nathalie Iannuccelli, Julien Sarry, Yvon Billon, Patrick Aymard, Virginie Helies, Cédric Cabau, Cécile Donnadieu, Julie Demars

**Affiliations:** 1 GenPhySE, Université de Toulouse, INRAE, ENVT, F-31326 Castanet-Tolosan, France; 2 GenESI, INRAE, F-17700 Surgères, France; 3 Sigenae, GenPhySE, Université de Toulouse, INRAE, ENVT, F-31326 Castanet Tolosan, France; 4 INRAE, GeT-PlaGe, Genotoul, F-31326 Castanet-Tolosan, France

**Keywords:** Rabbit, Targeted DNA sequencing, Capture, Exome, Human, Heterologous hybridization

## Abstract

Causal mutations for major genes that underlie a broad range of morphological traits are often located within exons of genes that then affect protein functions. Non-model organism genetic studies are not easy to perform due to the lack of genome-wide molecular tools such as SNP genotyping array. Genotyping-By-Sequencing (GBS) methods offer an alternative. Consequently, we used this approach that is focused on the exome to target and identify major genes in rabbit populations.

Data description

We used a heterologous enrichment method before sequencing, allowing us to capture the rabbit exome using the marketed human panel since mammal protein coding genes are well conserved across the phylogenic tree of species. This targeted strategy was performed on 52 French rabbits from 5 different French strains (Californian, New-Zealand, Castor, Chinchilla and Laghmere). We generated 3.4 billion sequencing reads and approximately 29–140 million of reads per DNA sample. The expected exome coverage per sample ranged between 118 and 566X.

The present dataset could be useful for the scientific community working on rabbit species in order to *(i)* improve the annotation of the rabbit reference genome Oryctolagus cuniculus (OryCun2.0), *(ii)* enrich the characterization of polymorphisms segregating in rabbits and *(iii)* evaluate the genetic biodiversity in different rabbit strains.

Raw sequences were deposited in the European Nucleotide Archive (ENA) at the European Molecular Biology Laboratory- European Bioinformatics Institute (EMBL-EBI) data portal under bioproject accession number PRJEB37917.

## Objective

Several studies have shown that 85% of causal mutations for major genes are located within exons [1]. However, non-model organisms often lack the molecular tools necessary to carry out genetic analyses for major gene identification or the molecular tools that exist are not informative enough. Although a medium density SNP array (Affymetrix AxiomOrcun Single Nucleotide Polymorphism Array, Thermo Fisher Scientific, USA) does exist for rabbit species, only major rabbit strains have been sequenced in order to develop and choose informative markers instead of cosmopolitan strains. In our study, both major (French Californian and French New-Zealand) and cosmopolitan (French Castor, French Chinchilla and French Laghmere) breeds were used. We therefore performed GBS as an alternative technology. We used a human targeted exome panel (Nextera Rapid Capture Exome, Illumina) that includes 241,126 human coding exons to capture and sequence rabbit exome. This heterologous hybridization approach has already been successfully carried out in canine species [2] and rabbit species is considered to be phylogenetically closer to human than dog [3]. Despite the poor quality of the current assembly of the reference rabbit genome (OryCun2.0, 7X coverage) and the version of annotation, in silico heterologous hybridization shows that 43.7% of the human probes uniquely match the OryCun2.0 reference genome that cover 114,225 annotated rabbit exons. Although the dataset is still confidential, preliminary analyses confirmed known exonic causal mutations as positive controls [4]**.**

Our raw dataset could be useful for the scientific community to improve the annotation of the rabbit reference genome since the current draft completed by the Broad Institute was built using a 7 × deep coverage. The exome enrichment method could possibly target and sequence novel rabbit exons. The read length and paired-end sequencing method allows a more accurate reads alignment with the reference genome. In addition, the quality and large coverage depth reads make it possible to identify novel variants in various rabbit populations especially for cosmopolitan breeds. Moreover, the present dataset could be useful for genetic biodiversity studies in rabbits.

Furthermore, this heterologous approach using a human molecular genetics tool could be used to study non-model organisms in mammalian species. The Whole Exome Sequencing (WES) method without custom panel is a rapid and low-cost alternative method to identify most exonic variants in a given species. This strategy could also be interesting for researchers who have a small quantity of biological material to carry out their genetics experiments.

## Data description

### Animals

The experiment included 52 French rabbits from 5 different strains that were all bred at INRAE experimental farm [5] or INRAE GenPhySE experimental facility in accordance with the French and European legislation on animal welfare. No animals in our study were bred/killed/taken specifically for the needs of our project, which therefore did not require explicit authorization (in accordance with the European Directive 2010/63/EU). The animals included 13 French Castors, 14 French Chinchillas, 20 French Laghmeres, 4 French Californians and 1 French New Zealand. Except for the French Californian and French New Zealand rabbits, strains were chosen for the morphological variability of their coat. French Castor, French Chinchilla and French Laghmere strains included 2 subgroups according to their coat phenotype for dilution (French Castor and French Chinchilla) [6] or angora (French Laghmere) [7]. French Californian rabbits were selected for feed efficiency [6] and the French New Zealand rabbit belong to a breed selected for reproduction traits [8]. All rabbits were adult animals whose sex and genealogy were known.

### Samples collection and DNA extraction

A total of 52 biological samples were collected from ear or skin biopsies or blood samples. Genomic DNA was extracted with an in-house protocol (protein K lysis followed by salt-based DNA extraction and ethanol precipitation), except for one sample that was extracted with the Dneasy tissue kit (Qiagen, Hilden, Germany). An additional extraction step was added for the seven blood DNA samples to first remove red blood cells. Total genomic DNA quality was determined using the Nanodrop 8000 spectrophotometer (ND8000LAPTOP, Thermo Fisher Scientific, USA) and the Fragment Analyzer (Advanced Analytical, USA) instrument. Total genomic DNA concentration was determined using the Qubit2.0 instrument (Q32866, Life Technologies, USA).

### Exome library preparation

A total of 53 exome libraries were prepared in 2 batches: one batch of 11 samples for a first proof of concept experiment, followed by a second batch of 42 samples under the same experimental conditions. Library preparations and exomes enrichment were performed using a human Nextera Rapid Capture Exome kit (version 1.2, Part#15037436 Rev. H, Illumina, USA). For one of the biological samples, two independent libraries were prepared, one in each batch. The protocol was performed according to the manufacturer's instructions, except for the second hybridization temperature that was set at 58 °C instead of 55 °C to accommodate heterologous hybridization of human probes to the rabbit genome. An additional purification (sizing AMPure XP 0,8X, Beckman Coulter) step was done to remove residual single-stranded probes. The average size of the library inserts was 378 bp and the concentration was more than 5 nM.

### DNA sequencing and raw data

DNA sequencing and Quality Control were performed in collaboration with the Genomic and Transcriptomic (GeT) core facility platform (INRAE, Toulouse, France, [9]). Fifty-three WES libraries were built on a HiSeq3000 sequencer in 2 × 150 bp paired-end reads (Hiseq3000 SBS kit 300 cycles, Illumina, USA). The whole dataset was sequenced on 6 lanes, 2 pools per lane on 5 lanes and 1 pool per lane on 1 lane (Flowcell HiSeq3000 8 lanes, Illumina, USA). Dual Indexes were used to demultiplex each sample with bcl2fastq (= CASAVA) software (version 1.8 or 2.20 depending on the batch). Libraries from the pilot batch were sequenced twice, while one sample (ERS4541894) was sequenced 3 times from 2 different libraries. Consequently, 64 raw sequences (fastq.gz files) were produced. Raw sequences were deposited in the ENA at EMBL-EBI ENA data portal under bioproject accession number PRJEB37917 [10]. We generated a total of 3.4 billion sequencing reads and approximately 29–140 million of reads per DNA sample. Given that the exome represents approximately 1% of the genome, the theorical exome coverage per sample ranged between 118X and 566X (Table 1).

**Table 1:** Overview of data set

Label	Name of data file/dataset	File types (file extension)	Data repository and identifier (DOI or accession number)
Dataset 1	Sequence raw data	Sequence file (.fastq.gz)	European nucleotide archive, https://identifiers.org/ena.embl:PRJEB37917, [8]

## Limitations

Expected coverage was evaluated with 100% heterologous hybridization. The quality of mapping and final coverage will be totally dependent on the quality of hybridization. Targeted exons depend both on the quality of the reference genome and the annotation of the species studied.

## Data Availability

The data presented is this Data Note can be freely and openly accessed on the EMBL-EBI ENA portal under bioproject accession number PRJEB37917, https://identifiers.org/ena.embl:PRJEB37917 [8].

## Abbreviations

GBS: Genotyping-by-sequencing
OryCun: *Oryctolagus cuniculus*
ENA: European nucleotide archive
EMBL-EBI: European molecular biology laboratory- european bioinformatics institute
SNP: Single nucleotide polymorphism
WES: Whole exome sequencing
GeT: Genomic and transcriptomic
